# Evaluating the buffering capacity of various soft drinks, fruit juices and tea

**DOI:** 10.4103/0972-0707.71643

**Published:** 2010

**Authors:** Smita Singh, Rahul Jindal

**Affiliations:** Department of Conservative and Endodontics, Darshan Dental College and Hospital, Ranakpur Road, Loyara, Udaipur, Rajasthan, India

**Keywords:** Buffering capacity, dental erosion, titratable acidity

## Abstract

**Aims and Objective:**

The purpose of this study is to measure the initial pH of various commonly used beverages and to determine their ability to maintain a low pH by measuring their buffering capacities.

**Materials and Methods:**

Twelve commercially available drinks were taken and divided into four groups (preserved fruit juices, tea, mineral water and carbonated drinks. Each group comprised of three drinks. Their initial pH were measured with pH meter and their buffering capacities were measured by adding 1M NaOH in the increments of 0.2 ml into 100 ml of each drink till the pH raised to 5.5 and 7 respectively.

**Statistical Analysis:**

The volume of NaOH required to raise the pH to 5.5 and 7 were recorded in all the groups. This data was subjected to statistical analysis using Mann- Whitney tests.

**Results:**

Total titratable acidity measurement shows that among all the drinks, there was no significant difference between carbonated drinks and preserved fruit juices while a significant difference was present between carbonated drinks, preserved fruit juices and tea.

**Conclusion:**

In this *in vitro* study, it was found that packaged apple juice had the most buffering capacity with maximum erosive potential whereas green tea had the least.

## INTRODUCTION

Dental erosion is defined as loss of tooth structure by a chemical process not involving any bacteria (Pindborg 1970).[1] Dental erosion is a prevalent problem everywhere. Soft drinks have been extensively investigated over a long period and are undoubtedly one of the principal factors in the aetiology of extrinsic dental erosion. Drinks that are consumed frequently are fruit juices, carbonated drinks and tea. The drink which causes erosion has low pH intrinsically.[2] The erosive potential depends on low pH and buffering capacity of the drinks. There are various acids that are present in beverages -carbonated drinks contain carbonic acid, packaged fruit juices contain organic acids derived from the fruits and the preservatives which are responsible for their erosive potential.[3] It has been accepted that titratable acidity which is a measurement of the total acid content, is a more important indicator than actual pH value in determining erosive potential of beverages.[4] The acid content of the drinks influence the buffering capacity and therefore the drinks that have low pH are strongly buffered with a potential erosive capability. The aim of this study was to measure the initial pH of various commonly used beverages and to determine their ability to maintain a low pH by measuring their buffering capacities.

## MATERIALS AND METHODS

For this *in vitro* study 12 commercially available drinks were tested: three varieties of preserved (FJ) fruit juices, three varieties of carbonated beverages (CD), three varieties of tea (T) and three varieties of mineral water (MW) which was taken as the control group.

Preserved fruit juices (FJ): This group comprised of 3 commercially available fruit juices like:

**Table:** 

Name of the drink	Trade name	Manufacturer
Apple juice	Appy fiz	Epicu Agro Products Pvt Ltd, India
Pineapple juice	Real pineapple fruit juice	Dabur foods Pvt Ltd, India
Orange juice	Real orange fruit juice	Dabur foods Pvt Ltd, India

Carbonated drinks (CD) This group comprised of 3 commercially available carbonated drinks:

**Table:** 

Name of the drink	Trade name	Manufacturer
Orange	Fanta	Coca Cola India Pvt Ltd, India
Lemon	Limca	Coca Cola India Pvt Ltd, India
Cola	Thums up	Coca Cola India Pvt Ltd, India

Tea (T This group comprised of 3 commercially available Tea:

**Table:** 

Name of the drink	Trade name	Manufacturer
Green tea	Twinning green tea	Twinnings Tea, London
Black tea	Twinning black tea	Twinnings Tea, London
Lemon tea	Lipton lemon tea	Lipton Tea, India

Plain mineral water (MW) This group comprised of 3 commercially available mineral waters taken as control group.

**Table:** 

Mineral water	Manufacturer
Bisleri	Parle India Pvt Ltd
Aquafina	PepsiCo India Pvt Ltd
Kinley	Coca Cola India Pvt Ltd

pH measurement: The initial pH of each drink was measured using a pH meter (Cyber Scan pH 620 by Eutech, India). 100ml of freshly opened drink at room temperature was placed in a beaker and stirred using a non heating magnetic stirrer until a stable reading was obtained. Three readings were taken of each drink from each group to give a mean measurement for that drink.

Buffering capacity: 100ml of each drink was titrated with 1M NaOH added in 0.2ml increments until the pH reached 5.5 and 7. This was done by using a non heating magnetic stirrer until a stable pH reading was obtained after each increment (0.2ml) of NaOH..This was done to measure the total titratable acidity (Touyz and Silove, 1993).[5] Titrations were repeated in triplicate for all drinks to check for reproducibility and to give a mean value for that drink.

Titratable acidity of a solution is measured by reacting the acids present with a base such as sodium hydroxide (NaOH) to a chosen end point, close to neutrality. The titratable acidity was kept at 5.5 and 7. The amount of NaOH required to raise the pH to 5.5 and 7 was noted and the data was subjected to statistical analysis using Mann Whitney test.

## RESULTS

### pH measurement

The pH and standard deviation values are shown in Table 1. The initial pH was lowest for carbonated drinks (3.33; d=0.18) and highest for tea (5.31; s.d.=0.71). According to this it can be stated that carbonated drinks were most acidic among all the beverages.

**Table 1:** Mean initial pH of various groups of beverages

Group	Code	Mean pH	S.D.
Preserved fruit juices	FJ	3.96	0.19
Carbonated drinks	CD	3.33	0.18
Tea	T	5.31	0.71
Mineral water	MW	7.34	0.12

SD - standard deviation

### Buffering capacity

Table 2 shows amount of NaOH needed to raise the pH of beverages to 5.5 and 7 respectively. Since green tea has an initial pH of 6.13, minimum amount of NaOH i.e. 0.1 ml was required to raise the pH to 7. Maximum amount of NaOH i.e. 4.4 ml was required to raise the pH of apple juice up to 5.5 and a total of 7.5 ml NaOH was required to raise the pH to 7. Hence it can be stated that more base was required for apple juice to neutralize its acidity.

**Table 2:** Titratable acidity of various beverages up to pH 5.5 and 7

Beverages	Initial ph	Titratable acidity (amount of NaOH req) up to pH 5.5	Titratable acidity (amount of NaOH req) up to pH 7.
Fanta	3.46	3.4 ml	6.8 ml
Limca	3.49	2.7 ml	5.7 ml
Thums up	3.16	2 ml	5.6 ml
Appy juice(apple)	3.78	4.40 ml	7.5 ml
Orange juice	3.96	4.9 ml	6.1 ml
Pineapple juice	4.16	5.7 ml	6.9 ml
Green tea	6.13	-	0.1 ml
Lemon tea	4.9	0.1 ml	0.5 ml
Black tea	4.89	0.2 ml	0.5 ml
Bisleri	7.27	-	-
Aquafina	7.31	-	-
Kinley	7.01	-	-

### Statistical analysis

Table 3 shows the *P* values for the individual comparisons made between each group of drinks for the number of ml of NaOH required to raise the pH to 5·5 and 7 respectively using Mann- Whitney test. In this study *P*<0.001was considered as significant.

**Table 3:** *P* values comparing the amount of NaOH in ml to raise the pH to 5.5 and 7

pH 5.5	CD	FJ	T
CD	-	NS	<0.001
FJ	NS	-	<0.001
T	<0.001	<0.001	-
pH 7.0	CD	FJ	T
CD	-	NS	<0.001
FJ	NS	-	<0.001
T	<0.001	<0.001	-

There was no significant difference between the preserved fruit juices and carbonated drinks at various pH levels. There was a significant difference between the carbonated drinks and preserved fruit juices in relation to tea.

## DISCUSSION

In 1970, Pindborg defined dental erosion as the irreversible loss of tooth structure due to chemical dissolution by acids and not of bacterial origin.[1] Erosion depends on several intrinsic and extrinsic factors. Acidic drinks and foods lower the pH level of oral cavity hence their consumption causes the teeth to demineralise[6] Erosion is found initially in the enamel and, if unchecked, may proceed to the underlying dentin. Even though pH is used to measure acidity yet the titratable acidity or buffering capacity, may truly indicate the potential of a beverage to erode tooth structure. The increased consumption of soft drinks has been linked to an increase in dental erosion but there is generally a widespread ignorance about the damaging effects of acid erosion due to packaged fruit juices. These are regarded healthy as natural fruit juices but because of the added preservatives they might have a higher buffering capacity. So they require more bicarbonate ions from saliva to get neutralized and hence have a greater erosive potential than other beverages. Intrinsic dental erosion is known as perimolysis, whereby gastric acid from the stomach comes into contact with the teeth. People with diseases such as anorexia nervosa, bulimia, and gastro-oesophageal reflux disease often suffer from this. There are many signs of dental erosion like sensitivity, transparent cutting edges with yellowish dentinal hue leading to discoloration due to loss of enamel. It has been well documented that the drop in pH of the oral cavity below critical pH i.e. 5-5.5, leads to demineralization of dental hard tissue leading to dental erosion.

The results of this *in vitro* study indicate that the drinks within any one group behave in a similar fashion due to their acid content. The buffering capacities of beverages tested *in vitro* can therefore be ranked as follows: Preserved fruit juices>carbonated drinks>tea. The fruit-based drinks and carbonated drinks were significantly different from tea. It was also interesting to note that initial pH value gave no indication of the underlying buffering capacity and, therefore the erosive potential of the drink. Generally, the preserved fruit juices had a higher initial pH than the carbonated drinks but required much more NaOH to raise the pH close to neutrality. This study agrees broadly with those already found in the literature[2,3] that fruit juices have greater erosive potential. This could be due to the addition of preservatives and flavouring agents which have a marked effect on the total acidity. However, the current investigation goes on to compare fruit juices with carbonated drinks and tea as they are consumed very commonly by Indian population.

Modification by adding calcium and phosphate to the drinks may be a helpful measure to reduce the erosive potential of the drinks. *In vivo* study should be carried out to ascertain whether the increased buffering properties of fruit based drinks have a greater potential to lower the pH at the tooth surface.[7]

## CONCLUSIONS

Under the limitations of this study it can be concluded that:

- There is no correlation between the initial pH of the drink and their erosive potential.
- Preserved fruit juices have significantly greater buffering capacity as compared to carbonated drinks and tea.
- Packaged apple juice had the most erosive potential whereas green tea had the least.
